# Correction to “Successful Treatment With Spesolimab in a Haemodialysis Patient With Acutely Flaring Generalised Pustular Psoriasis”

**DOI:** 10.1002/jvc2.70313

**Published:** 2026-03-05

Fujita, Y., Itamoto, S., Orita, A., Kawashima, K. and Shimizu, S. (2025), Successful Treatment of Spesolimab in a Haemodialysis Patient With Acutely Flaring Generalised Pustular Psoriasis, *JEADV Clinical Practice*, 4: 1190–1192. https://doi.org/10.1002/jvc2.70093.

In the Originally Published Article, the Title Contained a Grammatical Error. The Title:

“*Successful Treatment of Spesolimab in a Haemodialysis Patient With Acutely Flaring Generalised Pustular Psoriasis*.”

Has Been Corrected To:

“*Successful Treatment With Spesolimab in a Haemodialysis Patient With Acutely Flaring Generalised Pustular Psoriasis*.”

We apologize for this error.

